# RNA-sequencing elucidates the regulation of behavioural transitions associated with the mating process in honey bee queens

**DOI:** 10.1186/s12864-015-1750-7

**Published:** 2015-07-31

**Authors:** Fabio Manfredini, Mark J F Brown, Vanina Vergoz, Benjamin P Oldroyd

**Affiliations:** School of Biological Sciences, Royal Holloway University of London, Egham, TW20 0EX UK; Behaviour and Genetics of Social Insects Laboratory, School of Biological Sciences A12, University of Sydney, Sydney, NSW 2006 Australia

**Keywords:** *Apis mellifera*, Mating, Carbon dioxide, Double narcosis, Transcriptomics, RNAseq, Australian honey bees, Gene expression, Behaviour, Brain

## Abstract

**Background:**

Mating is a complex process, which is frequently associated with behavioural and physiological changes. However, understanding of the genetic underpinnings of these changes is limited. Honey bees are both a model system in behavioural genomics, and the dominant managed pollinator of human crops; consequently understanding the mating process has both pure and applied value. We used next-generation transcriptomics to probe changes in gene expression in the brains of honey bee queens, as they transition from virgin to mated reproductive status. In addition, we used CO_2_-narcosis, which induces oviposition without mating, to isolate the process of reproductive maturation.

**Results:**

The mating process produced significant changes in the expression of vision, chemo-reception, metabolic, and immune-related genes. Differential expression of these genes maps clearly onto known behavioural and physiological changes that occur during the transition from being a virgin queen to a newly-mated queen. A subset of these changes in gene expression were also detected in CO_2_-treated queens, as predicted from previous physiological studies. In addition, we compared our results to previous studies that used microarray techniques across a range of experimental time-points. Changes in expression of immune- and vision-related genes were common to all studies, supporting an involvement of these groups of genes in the mating process.

**Conclusions:**

Our study is an important step in understanding the molecular mechanisms regulating post-mating behavioural transitions in a natural system. The weak overlap in patterns of gene expression with previous studies demonstrates the high sensitivity of genome-wide approaches. Thus, while we build on previous microarray studies that explored post-mating changes in honey bees, the broader experimental design, use of RNA-sequencing, and focus on Australian honey bees, which remain free from the devastating parasite *Varroa destructor*, in the current study, provide unique insights into the biology of the mating process in honey bees.

**Electronic supplementary material:**

The online version of this article (doi:10.1186/s12864-015-1750-7) contains supplementary material, which is available to authorized users.

## Background

Mating is a key, and complex social behaviour, which is central to reproductive success across the animal kingdom. While mating behaviour has been the focus of numerous phenomenological studies (reviewed in [1]), there is growing interest in elucidating its molecular underpinnings in order to characterize its plasticity and evolution across animal taxa (e.g., [2, 3]). Brain transcriptomes can be used to associate complex behaviours like mating with patterns of gene expression at a genomic scale [2, 3], and to identify conserved molecular pathways across taxa [4, 5]. Such studies have revealed some of the specific molecules responsible for courtship or post-mating changes in behaviour in model organisms ranging from *Drosophila melanogaster* to voles [6–9]. However, the field of behavioural genomics is still in its infancy and further studies are needed to characterize the complexity and plasticity of mating behaviour in non-model organisms (see [10]) and to investigate the existence of genetic toolkits shared across taxa.

Social insects are likely to play a key role in developing our understanding of the molecular underpinnings of mating behaviour and post-mating changes. In social Hymenoptera (ants, some bees and wasps) queens only mate during a short period early in their life and undergo profound behavioural changes after mating, as they transform into nest-bound egg-laying machines. Mated queens have hugely extended lifespans relative to non-reproductive workers or similar solitary female insects [10–12]. Furthermore, because of the specialisation of reproductive and non-reproductive individuals in insect societies, the mating process may trigger sets of trade-offs that are different to those observed in non-social organisms. For example, the trade-off between reproduction and both immunity and longevity seen in solitary invertebrates [13, 14] may be uncoupled in social insects [10–12]. Consequently, studies of social insects may provide unique insights into the molecular mechanisms at play during and after the mating process. Thanks to modern genomic tools, such insights are emerging for ants [15, 16], wasps [17, 18], termites [19, 20], and honey bees [21, 22].

Honey bees (*Apis mellifera*) provide an excellent model system for molecular studies, as the behavioural ecology of mating is well understood. Queens mate on the wing when 6–13 days of age [23]. Virgin queens leave their nest during the afternoon [23], and may fly several kilometres [24] to mate at leks known as drone congregation areas [25]. At the lek they mate with multiple males in quick succession [26], before returning to their nest. About 50 % of queens commence oviposition after one mating flight, while the remainder fly on 1–2 subsequent afternoon(s) and again mate with multiple males [23, 27–29]. Physical stimulation of the *bursa copulatrix* during mating, as opposed to mating flights, contact with drones, or the presence of semen in the spermatheca, is responsible for activating oviposition [30]. Mating is associated with profound behavioural [31] and physiological [32–34] changes that are unrelated to the age of the queen. In particular, virgin queens are photophilic and eager to fly [31], and are aggressive towards other virgin queens [35]. In contrast, mated queens are photophobic, seek the protection of clustered workers and are unlikely to engage in fighting with other queens. Microarray studies have shown that these changes in behaviour and physiology are associated with profound differences in gene expression in the brain [22, 31].

Interestingly, similar physiological and behavioural changes to those found in mated queens are observed in queens subjected to double narcosis with carbon dioxide (CO_2_), providing an elegant tool for dissecting the molecular aspects of post-mating changes [36–40]. Insects possess specialized receptor cells that can detect and measure environmental CO_2_ [41, 42]. Honey bees use these receptors to help regulate CO_2_ concentrations in their nest by wing fanning [43–45]. Narcosis, caused by artificial CO_2_ exposure at much higher concentrations (50 % vs. a maximum of 3 % in the nest), usually triggers oviposition [46–48] by accelerating germ cell differentiation and stimulating the initial differentiation of the vitellarium [49]. It has been hypothesized that CO_2_ might achieve these results by exploiting the carbonic anhydrase pathways, the hypoxia-induced transcription factors or via activation of transferrin [50]. Narcosis can thus be used experimentally to separate the effects of insemination and mating flights from ovary activation on physiology and gene expression. Such studies have shown that treatment of honey bee queens with carbon dioxide stops mating flights, activates ovaries, alters the chemical profiles of mandibular glands, and affects gene expression both globally and for specific key genes such as *dopaminereceptors *[36, 50–52].

Here we examine the effects of the mating process and CO_2_-narcosis on gene expression in the brains of eight day old queen honey bees from an Australian population using RNA-sequencing (RNAseq). We expected CO_2_-treated queens to exhibit gene expression patterns intermediate between virgin and naturally-mated queens. Our study is an important step forward in understanding the relationship between mating and gene expression in honey bees. First, it directly compares the neurogenomics of virgin, CO_2_-treated and naturally-mated honey bee queens within the same study, enabling us to dissect how the mating process, as opposed to just the stimulation of egg-laying, changes gene expression. Second, it is the first RNAseq study of such gene expression, and thus builds on and complements previous studies [21, 22, 31, 50] that have used the informative but less-powerful microarray approach [53].

## Methods

### Biological material

In November 2012 we reared 20 sister queens of standard Australian commercial stock (primarily of Italian *A.m. ligustica* heritage) using standard beekeeping techniques from a single source colony [54]. The day before the young queens were due to emerge from their pupal cells we transferred them to an incubator at 35 °C and emerged them in individual glass vials [54]. At one day of age we paint-marked the queens and introduced each of them into their own nucleus colony. The entrance of each hive was covered with a queen excluder – a grid that allows workers to exit and enter, but confines the larger queens within the hive.

When the queens were 6 days old we randomly assigned the twenty queens to one of three treatments: *1) Mated queens* (*n* = 7) were queens that successfully participated in a mating flight. Early afternoon of the 6^th^ day, we removed the queen excluders, and then monitored the entrances until mating flights with successful mating (as indicated by a mating plug) were observed. *2) CO*_*2*_*-treated queens* (*n* = 7) were subjected to 10 min of CO_2_-narcosis on day 6 and on day 7. The seven caged queens were placed in a zip-lock plastic bag, which was then flushed with compressed CO_2_ until all queens were completely immobile, and sealed for 10 min. Queens were released in their individual colonies after treatment on both days. *3) Virgin queens* (*n* = 6) were caged along with the CO_2_-treated queens, but were returned to their respective colonies without narcosis.

All queens were harvested 2 days after treatment when they were 8 days old, at the same time of day (15:00 h), directly into liquid nitrogen and then stored at −80 °C. We used 4 queens per treatment group for RNAseq (total = 12 samples).

### Dissections and RNA isolation

Queens were stored at −80 °C prior to dissection. Abdomens were dissected to examine ovary activation and the presence or absence of semen in the spermatheca. All mated queens had activated ovaries (defined as possessing developing eggs) and semen in their spermathecae, while no eggs or semen were detected in virgin and CO_2_-treated queens. Head capsules were removed and brains were dissected over dry ice [55]. Brains were placed individually in Trizol and macerated to breakdown tissue. After addition of chloroform, samples were briefly vortexed and then centrifuged at 12,000 g for 15 min at 4 °C to generate an aqueous phase. After addition of isopropyl alcohol, the aqueous phase was again vortexed and centrifuged (10 min) to produce a pellet. The pellet was then washed with 75 % ethanol prior to drying and redissolving in ddH_2_O. The dissolved RNA was treated with DNAse I buffer (Ambion) to remove gDNA contaminations, prior to centrifugation and collection of the aqueous phase.

The RNA content of the sample and the purity of the extracts were assessed using a NanoDrop™ 1000 spectrophotometer (Thermo Scientific Inc., Bremen, Germany).

### RNA-sequencing

We sequenced RNA with an Illumina HiSeq system using 2 lanes of a plate (6 samples per lane) and producing 50 bp single-end reads. Reads were checked with FastQC (http://www.bioinformatics.babraham.ac.uk/projects/fastqc/) for quality control and were subsequently processed with Trimmomatic [56] to remove adapters and low quality bases: reads less than 36 bases long were filtered out (Additional file 1). We also dropped reads that matched ribosomal RNA sequences (rRNA) by means of SortMeRna [57]. Surviving reads were aligned with TopHat for Illumina [58] to the latest version of the honey bee genome (*Apis mellifera* Assembly 4.5) available on BeeBase (http://hymenopteragenome.org/beebase/?q=download_sequences) by using the Galaxy web-based platform (https://usegalaxy.org/). Mapped reads were converted into raw read counts with SAMtools idxstats [59] and these were used to quantify differential gene expression.

### Analysis of gene expression

Raw read counts were analysed with R using the edgeR package from Bioconductor [60]. Read counts were log_2_ transformed to correct for the skew to zero and large values. Only genes with at least 10 reads per sample were kept in the analysis (12,992 genes). Normalization was performed with Trimmed Mean of M-values (TMM), a method that is implemented in the edgeR Bioconductor package [61]. We detected differential levels of gene expression using a modified Fisher’s exact test that takes into account both dispersion and multiple samples. Finally, raw *P*-values for each gene were corrected for multiple comparisons setting a false discovery rate (FDR) of 5 %.

For global analyses of gene expression we used hierarchical clustering (Ward method) and principal component analysis in JMP Pro 10.0 (SAS, Cary, NC). To perform Gene Ontology (GO) analyses we obtained *Drosophila melanogaster* orthologs with BLAST (http://blast.ncbi.nlm.nih.gov/Blast.cgi) for honey bee genes that were significantly differentially expressed between treatments and computed functional annotation clustering in DAVID version 6 [62, 63] with medium stringency and a cutoff of *P*-value < 0.05. To identify overrepresented biological functions (enrichment analysis) we compared the annotation composition in our list of differentially expressed genes to that of a population background composed of all the honey bee genes with *Drosophila* orthologs.

### Comparative studies

We used Venny (http://bioinfogp.cnb.csic.es/tools/venny/index.html) to overlap *D. melanogaster* ortholog matching lists of significantly differentially expressed genes and a Hypergeometric test (http://nemates.org/MA/progs/overlap_stats.html) to assess whether genes overlapping between studies occurred significantly more often than expected by chance. We compared our study to three microarray studies [22, 31, 50] and one qPCR study [64] on honey bee gene expression after either mating or CO_2_-narcosis (for more details see Results and Additional files 2, 3, 4, 5, 6, 7). For comparisons with microarray studies, we first overlapped whole datasets from each study to identify potential candidate genes for mating behaviour and response to carbon dioxide and performed GO analysis on them. We also performed overlap analyses on genes from pairwise comparisons of interest to evaluate the level of agreements across studies.

## Results

### RNA-sequencing

The sequencing produced 30 million reads per sample on average (min 22,298,993 and max 39,301,839, see Additional file 1). About 7 % of total reads were dropped during the filtering steps and a further 4 % were excluded as ribosomal RNA. On average, 98 % of surviving reads per sample were aligned to single locations in the honey bee genome.

### Global analysis of gene expression

**Fig. 1:**
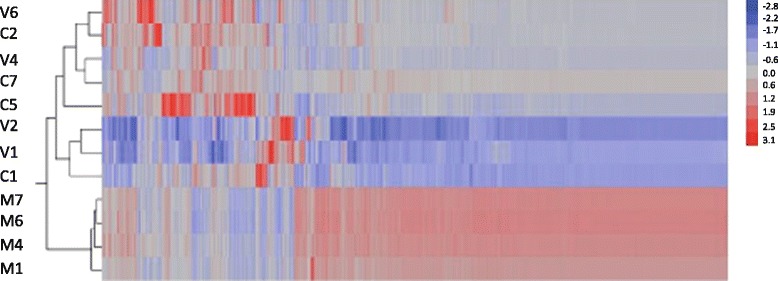
Patterns of gene expression in individual queens. The Heatmap was obtained after hierarchical clustering analysis of log_2_-transformed and normalized read counts that corresponded to 1088 genes significantly differentially expressed across treatments. The analysis shows that mated queens cluster separately as compared to virgin and CO_2_-treated queens. Genes in red are significantly up-regulated while genes in blue are significantly down-regulated. V = virgin, M = mated, C = CO_2_-treated

A total of 1088 genes were significantly differentially expressed (FDR < 0.05) in at least one of three pairwise comparisons across treatments (see below); this represents 7.10 % of the 15,314 coding sequences present in the honey bee genome. We performed a hierarchical clustering analysis on this set of genes to identify common patterns of gene expression across individual samples. We obtained a clear separation of our samples into two groups: mated queens clustered alone while virgin queens and CO_2_-treated clustered together forming a separate macro-group (Figure 1). This suggests that the mating treatment was the major driver of gene expression in the honey bee brain.

**Fig. 2:**
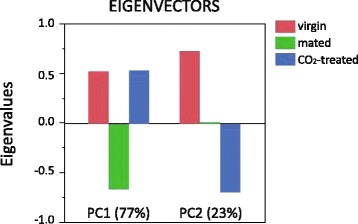
Multivariate analysis of gene expression after mating and double narcosis with CO_2_. The principal component analysis of 1088 significantly differentially expressed genes shows that the difference was primarily due to the mating process (77 %) and to a minor extent the CO_2_ treatment (23 %)

We also performed a principal component analysis on the same set of 1088 significantly differentially expressed genes (Fig. 2). Again, most of the changes in gene expression (77 %) were associated with the mating process: mated queens clustered on the opposite side of the chart compared to virgin and CO_2_-treated queens. Only 23 % of the difference in gene expression was associated with CO_2_-treatment as compared to virgin queens.

### Pairwise comparisons between treatments

#### Mated vs. virgin queens

**Fig. 3:**
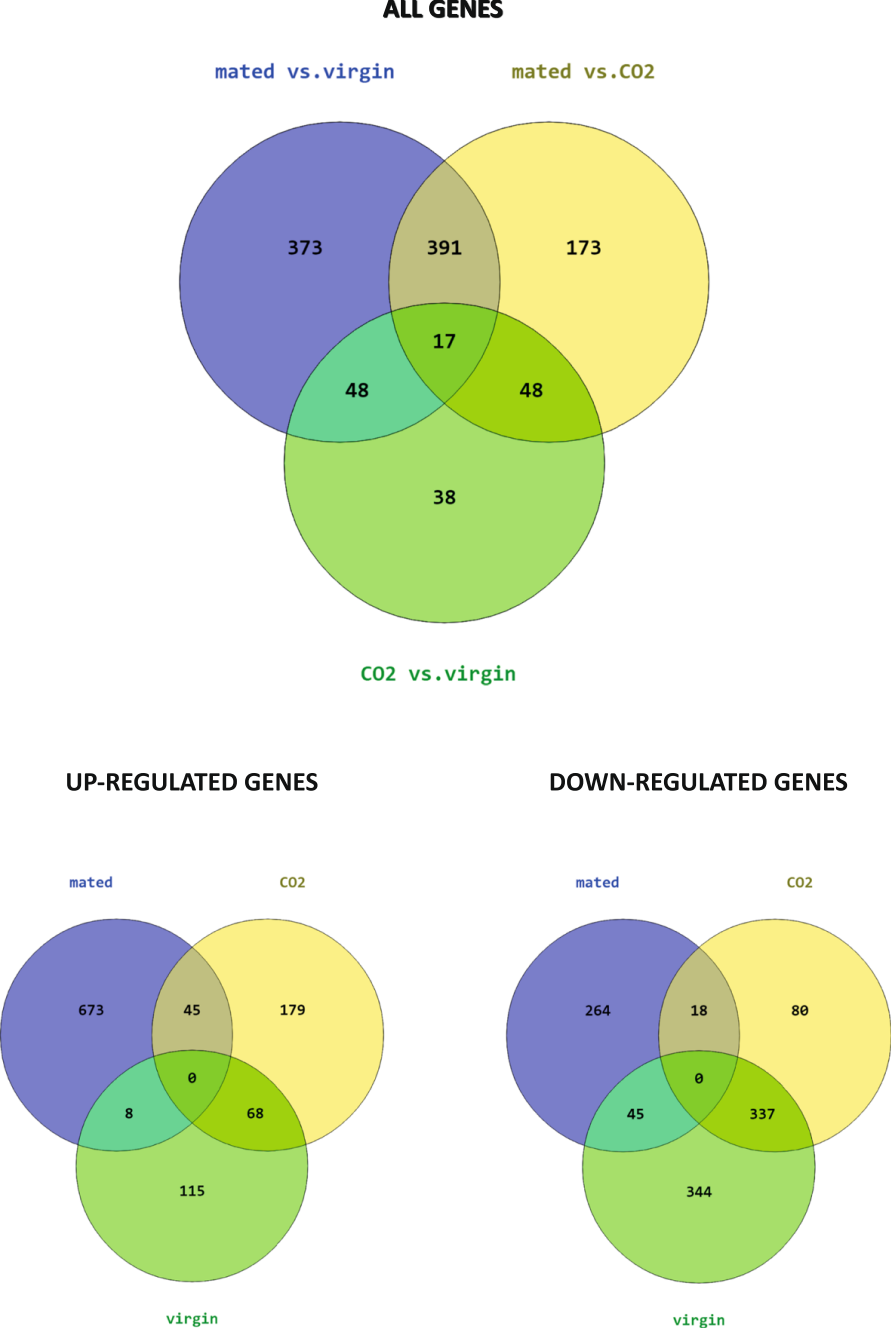
Pairwise comparisons of gene expression between treatments. ALL = genes that were significantly differentially expressed between treatment groups. UP = total numbers of unique genes that were up-regulated across all comparisons for each treatment group. DOWN = total numbers of unique genes that were down-regulated across all comparisons for each treatment group

Pairwise analysis of gene expression between treatments revealed a large set of genes that were significantly differentially expressed between mated and virgin queens: 829 genes at FDR < 0.05 (Fig. 3 and Additional file 8). Of these, 654 genes were up-regulated and 175 were down-regulated in mated queens. Our GO analysis was based on the 475 *D. melanogaster* orthologs for these genes. Six metabolic pathways and 28 GO terms were significantly overrepresented (Table 1 and Additional file 9). Of particular interest were GO terms associated with the following key biological processes: sensory perception (*P*-value = 0.011*,* 15 genes, up-regulated in mated = 5, down-regulated = 10), detection of stimulus (*P*-value = 0.040, 8 genes, up-regulated in mated = 2, down-regulated = 6), multiple metabolism-related GO terms such as the fatty acid metabolic processes (*P*-value =0.015, 7 genes, all up-regulated in mated) and the immune-related GO terms defense response (*P*-value = 0.009, 11 genes, up-regulated in mated = 10, down-regulated = 1), melanisation defense response (*P*-value = 0.012, 3 genes, all up-regulated in mated) and innate immune response (*P*-value = 0.015, 7 genes, up-regulated in mated = 6, down-regulated = 1). See Additional file 9 for a list of the genes associated with these GO terms.

**Table 1:** Summary of Gene Ontology Analysis of differentially expressed gene lists. The analysis is based on *D. melanogaster* orthologs matching honey bee genes that were significantly differentially expressed between treatment groups. Only GO terms and KEGG pathways that were significantly overrepresented (medium stringency, *P*-value < 0.05) are shown in the table. Benjamini corrections for multiple testing are provided alongside corresponding *P*-values

	Category	Term	Genes	*P-*Value	Benjamini
mated vs. virgin		carbohydrate metabolic process	30	*3.33E-05*	0.022
		organic acid metabolic process	27	*1.79E-04*	0.076
		amine metabolic process	26	*1.00E-03*	0.209
		defense response	11	*0.009*	0.778
		sensory perception	15	*0.011*	0.782
		melanization defense response	3	*0.012*	0.764
	biological process	cellular amino acid metabolic process	16	*0.015*	0.76
		fatty acid metabolic process	7	*0.015*	0.737
		innate immune response	7	*0.015*	0.737
		carbohydrate catabolic process	8	*0.017*	0.752
		tissue development	30	*0.026*	0.805
		detection of stimulus	8	*0.04*	0.805
		sodium ion transport	6	*0.041*	0.794
	cellular component	myofibril	5	*0.002*	0.035
		membrane part	81	*0.003*	0.046
		proteinaceous extracellular matrix	7	*0.006*	0.071
		plasma membrane	39	*0.009*	0.093
		cofactor binding	19	*7.91E-05*	0.022
		monooxygenase activity	12	*0.002*	0.151
		lipase activity	10	*0.004*	0.214
		FAD binding	8	*0.005*	0.227
		vitamin binding	10	*0.008*	0.259
	molecular function	sugar transmembrane transporter activity	5	*0.014*	0.29
		transcription factor activity	23	*0.018*	0.325
		receptor activity	27	*0.018*	0.318
		transforming growth beta receptor binding	3	*0.023*	0.336
		identical protein binding	11	*0.03*	0.368
		sulfate transmembrane transporter activity	3	*0.037*	0.424
	metabolic pathways	Starch and sucrose metabolism	7	*0.003*	0.081
		Glycolysis / Gluconeogenesis	7	*0.004*	0.061
		Retinol metabolism	5	*0.006*	0.058
		Arginine and proline metabolism	7	*0.006*	0.051
		beta-Alanine metabolism	5	*0.013*	0.087
		Lysine ddegradation	5	*0.05*	0.192
mated vs. CO_2_	biological processes	aromatic amino acid family process	5	*0.003*	0.757
		sensory perception	11	*0.024*	0.995
		monocarboxylic acid metabolic process	7	*0.031*	0.992
		tissue development	22	*0.032*	0.987
		response to organic substance	8	*0.044*	0.992
	cellular component	membrane	70	*1.00E-03*	0.014
		muscle myosin complex	3	*0.004*	0.053
		extracellular region part	8	*0.032*	0.255
	molecular function	sugar transmembrane transporter activity	5	*0.006*	0.222
		transporter activity	33	*0.007*	0.246
		retinal binding	3	*0.018*	0.394
		lipase activity	6	*0.033*	0.545
		olfactory receptor activity	5	*0.037*	0.551
CO_2_ vs. virgin	biological process	cognition	7	*0.004*	0.105
	cellular component	rhabdomere	4	*1.00E-03*	0.01
		membrane	21	*0.001*	0.012
		cell projection	5	*0.004*	0.039
		integral to membrane	12	*0.056*	0.333
	molecular function	monooxygenase activity	5	*3.09E-04*	0.054

#### Mated vs. CO_2_-treated queens

There were 629 significantly differentially expressed (FDR < 0.05) genes between mated and CO_2_-treated queens (Fig. 3 and Additional file 10): 409 genes were up-regulated and 220 were down-regulated in mated queens. GO analysis of the matching 310 *D. melanogaster* orthologs revealed that 13 GO terms were significantly overrepresented (Table 1 and Additional file 11), including sensory perception (*P*-value = 0.024*,* 11 genes, up-regulated in mated = 4, down-regulated = 7) and response to organic substance (*P*-value = 0.044, 8 genes, up-regulated in mated = 5, down-regulated =3). See Additional file 11 for a list of the genes associated with these GO terms.

#### CO_2_-treated vs. virgin queens

A much smaller set of 151 genes were significantly differentially expressed (FDR < 0.05) between CO_2_-treated and virgin queens (Fig. 3 and Additional file 12): 117 genes were up-regulated and 34 were down-regulated in CO_2_-treated queens. The 59 *D. melanogaster* orthologs for these genes produced 6 significantly overrepresented GO terms (Table 1 and Additional file 13), including the biological process cognition (*P*-value = 0.004*,* 7 genes, up-regulated in CO_2_-treated = 1, down-regulated =6). See Additional file 13 for a list of the genes associated with this GO term.

### Comparative studies

In order to compare our RNAseq analysis of the honey bee mating process and CO_2_-treatment to previous research addressing similar questions with the microarray technique, we overlapped lists of significantly differentially expressed genes from our study and the three following studies: 1) Kocher et al. [22], where the authors examined brain gene expression in virgin, mated and egg-laying honey bee queens, providing obvious potential comparisons with our mated queens; 2) Kocher et al. [31], where they examined the effects of mating and instrumental insemination with saline or semen on gene expression in the brains of honey bee queens, providing useful comparisons with both our mated and CO_2_-treated queens (as instrumental insemination involves CO_2_-treatment); 3) Niño et al. [50], where the brain transcriptomic profile was evaluated in virgin, CO_2_-treated, and physically manipulated honey bee queens (i.e., exposed to CO_2_ and sham-inseminated), providing valuable comparisons for our CO_2_-treated queens.

**Fig. 4:**
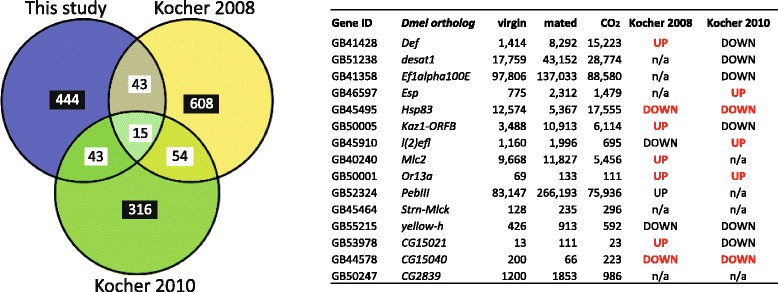
Best candidate genes for mating process and reproductive activation. Patterns of expression for 15 genes that were significantly differentially expressed in our study and in Kocher et al. [22] and Kocher et al. [31] (see Additional file 7). The table shows the total read counts for each gene in virgin, mated and CO_2_-treated queens from our study plus the direction of expression in mated vs. virgin queens in the other two studies: UP = up-regulated in mated queens; DOWN = down-regulated; bold red = same pattern of expression between studies; n/a = difference in gene expression occurs in a comparison which is not relevant for our study

**Fig. 5:**
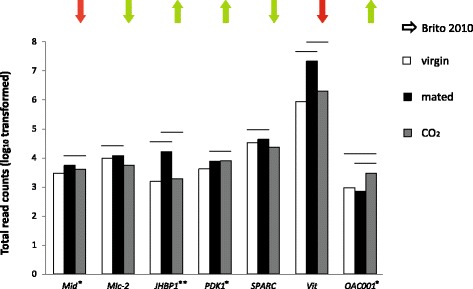
Response to carbon dioxide. Patterns of expression of 7 genes that were included in Brito et al. [64] and were also significantly differentially expressed in our study. Lines above bars indicate the pairwise comparisons where the genes were significantly differentially expressed (*P*-value < 0.05). Arrows show direction of expression for Brito et al. [64] (brains of CO_2_-treated vs. untreated 8-day-old queens): green = same trend as in our study; red = opposite trend. See Additional file 7 for more information on this comparative study

Very few genes were shared across studies for the focal pairwise comparisons, resulting in a lack of statistically significant overlap (see Figs. 4 and 5 and Additional files 2, 3, 4, 5, 6, 7 for details on these analyses). In contrast, at the whole dataset level, a significant number of differentially expressed genes were shared between our study and Kocher et al. [31]. However, no significant overlap was found in comparisons with data from Kocher et al. [22] and Niño et al. [50] (see Additional files 2 and 7). At the coarser GO level of analysis, immune-related GO terms and the GO term ‘response to other organism’ were recurrent across all queen studies (Kocher et al. [22, 31], Niño et al. [50] and this study).

## Discussion

The mating process and CO_2_-induced narcosis significantly affect the neurogenomic profile of honey bee queens, by changing the expression of more than a thousand genes within two days of treatment. The majority of these differences were driven by the mating process, maturation than natural mating. Importantly, changes in pathways related to vision, metabolism, chemoreception, and immunity match expectations from known behavioural changes. These next-generation sequencing results partially corroborate previous microarray studies, and provide new insight into the molecular regulation of key behavioural transitions in honey bee queens.

### Neurogenomics of mating behaviour

Gene expression in the brains of mated queens differs strongly from that seen in virgins. Strikingly, genes that are associated with vision such as *Rhodopsin 2* (*Rh2*), *neither inactivation nor afterpotential A* (*ninA*), *Arrestin 2* (*Arr2*), *G protein beta-subunit 76C* (*Gβ76C*), *chaoptin* (*chp*) and *Calhotin* (*Cpn*) were all down-regulated in mated queens compared to virgins (see Additional file 6). Changes in the expression of these visual perception genes mirrors the transition from photophilic behaviour observed in virgin queens that engage in mating flights, to more photophobic behaviour in mated queens confined within the nest. Queens are required to fly during swarming events [65], and it would be interesting to see if the vision system is reactivated in queens as they prepare to swarm. It would also be interesting to determine whether queens of open nesting honey bee species like *Apis florea*, in which the queens are able and ready to fly at all times [66], show the same decline in vision-related genes after mating.

Another group of genes that are differentially expressed in mated and virgin queens belong to the family of odorant receptors (ORs) and odorant binding proteins (OBPs). Odour and pheromone perception are central to the social life of honey bees [67–69] and play an important role in mate location and efficient mating. Of particular interest is the presence of the *Pheromone-binding protein-related protein 2* (*Obp19d*), which was down-regulated in mated queens. This gene is involved in the detection of pheromones, which are chemical compounds released by one individual to trigger a social response in members of the same species and therefore are widely used among social insects to mediate mating behaviour. This protein may be used by virgin honey bee queens to locate rival virgins. Interestingly, of ten genes encoding predicted pheromone or odorant binding proteins that are affected by mating in *Drosophila* [70] only four were also significantly differentially expressed in our study (*Obp19d, Obp56g, Or43a* and *Or49b*).

As previously reported [22, 31, 50, 71], genes broadly related to metabolism were also differentially expressed between mated and virgin queens (see Additional file 9). Mating flights are energetically expensive [72] whereas nest-bound life is not. It is therefore unsurprising that genes related to carbohydrate metabolism, glycolysis and gluconeogenesis were differentially regulated in mated queens relative to virgin queens. In contrast, the requirement for fatty acid metabolism increases after mating, because novel lipids are required for pheromone synthesis and egg production [73]. As expected, all seven genes in this cluster were up-regulated in mated queens.

Finally, the last important group of genes that differ between mated and virgin queens is the immune genes. Three of these genes, *Serine Protease Immune Response Integrator (spirit)*, *Peptidoglycan recognition protein SA (PGRP-SA)* and *Gram-negative bacteria binding protein 1 (GNBP1)* are important players in the *Toll* pathway and their expression is usually triggered by a challenge from Gram-positive bacteria [74]. Three additional genes, *Melanization Protease 1 (MP1)*, *Serpin-27A (Spn27A)* and *Hemolectin (Hml)* are involved in the melanisation response [75, 76] and can therefore be triggered by bacterial infection or wound healing reactions. Finally, two other genes in this group, the antimicrobial peptides *defensin* and *serpent*, are involved in hematopoiesis [77]. With the exception of *defensin*, all immune genes were up-regulated in mated queens and this is likely to result in higher immunocompetence as more defense molecules, such as antimicrobial peptides, are produced, and cellular responses or wound healing reactions may be more effective. Increased expression of immune genes post mating has been observed repeatedly in honey bees [22, 31, 50, 71] and other organisms [78]. In *Drosophila*, for example, 19 immune-related genes respond to mating, including *defensin* and *PGRP-SA* [70]. One explanation for up-regulation of immune genes in females after mating might be activation of the immune system by immune elicitors associated with the male reproductive apparatus or the ejaculate, or by a traumatic insemination event that leads to wound healing reactions. However, this does not seem to be the case in the honey bee, where it has been previously observed that non-traumatic instrumental insemination with sterile saline is sufficient to up-regulate the expression of immune genes [71]. Consequently, up-regulation of immune genes may be triggered, not by the physical process of mating, but through molecular cross-talk between reproductive and immune gene-expression pathways. Such cross-talk may have been selected because of adaptive advantages to the queen and to the colony, such as increased protection from horizontally transmitted parasites and pathogens [79]. In contrast, in solitary species immunocompetence is reduced after mating, so that energy can be redirected from the immune system to reproductive activity, i.e., sperm storage or egg-laying [80, 81]. Our results demonstrate that this type of trade-off is not present in honey bees, perhaps because the newly-mated queen can rely on the social immunity conferred by her natal colony, and because queens are fed a near-perfect diet of worker mandibular secretions [82, 83]. It would be interesting to conduct a comparative study across solitary- and swarm-founding social insects, to test this hypothesis. Another explanation for the observed pattern could be that virgin queens reduce their investment in immune defence in preparation for mating flights: this would allow them to allocate a greater portion of their energy resources in two activities (flying and mating) that are energetically expensive. A time-course study on the levels of expression of immune genes in honey bee queens from emergence to full reproduction would address this question.

In addition to the GO analyses, we examined the expression of several genes that might play a role in mating and ovary activation. Genes of the insulin/insulin-like signalling (IIS) and TOR pathways are responsible for regulating growth and nutrition and are fundamental to the process of honey bee caste determination [84]. We found that only one gene in these pathways was significantly differentially expressed between mated and virgin queens, *Phosphoinositide-dependent kinase 1* (*PDK1*). This gene was up-regulated in mated queens. The fact that *PDK1* was up-regulated in the only group of queens with activated ovaries in our studies is in line with the hypothesis that *PDK1* activity is linked to ovary size in worker bees, where foragers with a bias toward pollen have both larger ovaries and higher levels of expression of *PDK1* compared to foragers that are nectar-biased [85].

We also examined expression of the biogenic amines, as these compounds may be involved in mediating the interactions between brain and ovaries during reproductive activation. Dopamine signalling pathways are positively associated with reproductive status in workers [37, 40, 86]. For example, the gene *N-acetyldopamine* is positively correlated with ovarian development [87]. In queens, instead, dopamine and reproductive status are negatively correlated and dopamine levels decrease after mating [88]. Our study provides further support for this reversed relationship in queens, as *dopamine N-acetyltransferase* (*Dat*), a component of the catabolism of dopamine, was down-regulated in mated queens.

### Neurogenomics of CO_2_-narcosis

The most important differences in gene expression profiles between CO_2_-treated and virgin queens relate to cognition (see Additional file 13 for other differentially expressed genes). Most of the genes in this group were the same genes found in the GO terms “sensory perception” and “detection of stimulus” for the mated vs. virgin queen contrast; in addition we found *neither inactivation nor afterpotential C (ninaC)* and *no receptor potential A (norpA,* see Additional file 6). All cognition genes but one were down-regulated in CO_2_-treated queens, as they were in mated queens. The similarity in patterns of expression of these genes suggests that CO_2_-treated queens undergo a process of de-activation of visual perception and eye development genes similar to that seen in mated queens. One potential explanation for this is that CO_2_-narcosis induces acidosis, as may mating flights [89], indicating a potential role for body pH as a trigger for these changes in gene expression. However, this must be separate from the processes that link CO_2_-treatment to oviposition, as Koeniger et al. [30] have demonstrated that flight is not sufficient to induce oviposition in honey bee queens. This suggests that different aspects of the mating process may trigger distinct sets of changes in gene expression.

Despite such similarities in gene expression in mated and CO_2_-treated queens when compared to virgins, a direct comparison of the two groups also highlighted interesting differences. Expression of genes related to sensory perception differed strongly across the two groups, as did genes associated with olfactory and gustatory activity. Five ORs (*Or13a*, *Or43a*, *Or49a*, *Or85b* and *Or85c*) and *Obp19d* were up-regulated in CO_2_-treated queens, while three other OBPs (*Obp56d*, *Obp56g* and *Obp83b*) were up-regulated in mated queens. CO_2_-treated and mated queens also differ for genes related to the response to organic substances: among these, of particular relevance is the *Insulin-like receptor* (*InR*), which was down-regulated in CO_2_-treated queens. *InR* is a major player in the insulin signalling pathway and regulates important biological functions such as metabolism, growth, reproduction, and aging [90]. Again, this supports the idea that CO_2_-narcosis induces only a subset of the changes in gene expression caused by the process of mating and onset of oviposition. In addition, the two-day post-treatment time interval prior to sampling did not allow for complete development of eggs in CO_2_-treated queens, while it was sufficient for mated queens, as shown by our dissections. This is not surprising, as egg development has been observed after twelve days from narcosis or instrumental insemination [50, 71], which is a much longer time span. Together with our gene expression results, this confirms that mating and CO_2_-narcosis, despite producing the same final result, follow apparently different pathways of action.

### Comparative studies

Only two of the three whole dataset comparisons with previous studies produced statistically significant overlaps. However, there is a common pattern in the biological functions that are associated with genes that are shared between studies. GO terms related to immune functions and response to other organism are overrepresented across studies [22, 31, 50]. This makes us confident that these processes are key to the physiological and behavioural changes that take place as a queen transitions from her initial virgin state to that of a mated matriarch.

Interestingly, while the findings of Kocher et al. [31] are significantly congruent with our study, those from Kocher et al. [22] and Niño et al. [50] are not, no doubt as a consequence of the similarities and differences across the studies in experimental treatments. In our study and in Kocher et al. [31], queens were collected two days after treatment; in contrast, Kocher et al. [22] and Niño et al. [50] analysed samples collected 5 and 10 days after treatment, respectively. This suggests both that the neurogenomic state of an individual is repeatable across studies, and that it is temporally dynamic. Future studies are needed to understand how and why gene expression changes over time, both before and after mating.

Finally, a number of other factors may explain the low-level of congruence between our results and earlier studies. An obvious methodological factor is that, while previous studies used microarrays, we used RNAseq to generate transcriptomic data. RNAseq is a more powerful technique than microarrays, and relies on a different experimental/statistical approach (for a comparison between microarray and RNAseq platforms see Guo et al. [53]), which may hinder comparisons among studies. From a biological perspective, in all studies experimental bees were produced by a single colony. This approach is frequently used in studies of social insects, as it eliminates variance related to inter-colonial variability, resulting in higher statistical power to detect inter-individual differences in gene expression as a result of treatments applied to individual bees. However, such benefits are lost when comparing across studies, as each colony is a unit on its own (the super-organism) characterized by a particular social environment.

## Conclusions

This study is an important advance in the molecular characterization of the mating process in the honey bee *Apis mellifera*, a model system for sociobiology and the most important managed crop pollinator. Our results are partially in line with previous studies, but demonstrate interesting mismatches, denoting the importance of consistent experimental design in genomic studies. By uncovering many important biological functions associated with the mating process, this study also stresses once more the complexity of this behaviour and the need in the future to isolate the single components (e.g., mating flights and copulation) and analyse them separately. In addition, this study focused on Australian honey bees, which comprise one of the last populations free of the invasive parasitic mite *Varroa destructor* [91–93]. As such, our results provide a biological baseline that can be used as a reference to understand the impact of external challenges on bee decline in the Western world [94]. At the same time, this important difference in parasite load between populations of honey bees could underlie the differences in patterns of gene expression revealed by our comparative analyses. Future studies need to focus on determining the mechanisms behind cross-study variation to isolate key and repeatable differences in gene expression during the mating process in honey bees, as well as the impact of important parasites and pathogens on this process.

## Availability of supporting data

RNAseq raw sequence reads and normalized expression values for each gene are available in the NCBI Gene Expression Omnibus repository, Series record GSE65833 http://www.ncbi.nlm.nih.gov/geo/query/acc.cgi?token=oludywiunlwblgd&acc=GSE65833. Other data sets supporting the results of this article are included within the Additional files.

## Ethics

For this study honey bees from commercial colonies were used and therefore no ethics statement is required.

## Additional files

Additional file 1:
**Summary statistics for Illumina HiSeq, read preparation and alignment to the honey bee genome.**
Additional file 2:
**Comparative studies.** Overlap analyses with previous microarray studies on the effect of mating and CO_2_-narcosis on gene expression in honey bees. Additional file 3:
**Overlap analysis with Kocher et al. [** 22]. Genes that were significantly differentially expressed between mated and virgin queens in both studies. Additional file 4:
**Overlap analysis with Kocher et al. [** 31]. Genes that were significantly differentially expressed between mated and virgin queens in both studies. Additional file 5:
**Overlap analysis with Niño et al. [** 50]. Genes that were significantly differentially expressed between CO_2_-treated and virgin queens in both studies. Additional file 6:
**Genes involved in eye development and visual perception that were differentially expressed between treatments in this study.**
Additional file 7:
**Results of comparative studies.**
Additional file 8:
**Pairwise comparisons.** List of the genes that were differentially expressed between mated and virgin queens at FDR < 0.05. Additional file 9:
**Gene ontology analysis for the pairwise comparison mated vs. virgin.** Significantly enriched GO terms and KEGG pathways (functional annotation chart, P-value < 0.05) and details on associated genes for terms of interest. Additional file 10:
**Pairwise comparisons.** List of the genes that were differentially expressed between CO_2_-treated and mated queens at FDR < 0.05. Additional file 11:
**Gene ontology analysis for the pairwise comparison CO**_**2**_ **-treated vs. mated.** Significantly enriched GO terms (functional annotation chart, *P*-value < 0.05) and details on associated genes for terms of interest. Additional file 12:
**Pairwise comparisons.** List of the genes that were differentially expressed between CO_2_-treated and virgin queens at FDR < 0.05. Additional file 13:
**Gene ontology analysis for the pairwise comparison CO**_2_ **-treated vs. virgin.** Significantly enriched GO terms (functional annotation chart, *P*-value < 0.05) and details on associated genes for term of interests.

## Abbreviations

RNA: Ribonucleic acid
CO_2_: Carbon dioxide
RNAseq: High-throughput RNA sequencing
gDNA: genomic Deoxyribonucleic acid
ddH_2_O: double-distilled water
rRNA: ribosomal RNA
FDR: False discovery rate
qPCR: quantitative real-time polymerase chain reaction
GO: gene ontology
OR: Odorant receptor
OBP: Odorant binding protein
IIS: Insulin/insulin-like signaling pathway
TOR: Target of rapamycin pathway
KEGG: Kyoto encyclopedia of genes and genomes
