# Supplementary material for: RNA-sequencing elucidates the regulation of behavioural transitions associated with the mating process in honey bee queens
Source: BMC Genomics. 2015 Jul 31;16:563. doi: 10.1186/s12864-015-1750-7 (PMC4521450; doi:10.1186/s12864-015-1750-7)
Supplement: Additional file 6: — Genes involved in eye development and visual perception that were differentially expressed between treatments in this study. [file 12864_2015_1750_MOESM6_ESM.pdf]

Additional file 6. Genes involved in eye development and visual perception that were differentially expressed between treatments in our study.

Gene name	Symbol	Gene function	Reference
Arrestin 2	*Arr2*	**works with rhodopsins in the maintenance of photoreceptor**	[1]
Calphotin	*Cpn*	**involved in the development of the rhabdomeres**	[2]
chaoptin	*chp*	**involved in the development of the rhabdomeres**	[3]
G protein beta-subunit 76C	*Gp76C*	**responsible for terminating the active state of light response signalling cascade**	[4]
neither inactivation nor afterpotential A	*ninA*	**required for the biogenesis of rhodopsins**	[5]
neither inactivation nor afterpotential C	*ninaC*	**encodes a rhabdomere specific protein and is required to prevent retinal degeneration**	[6]
no receptor potential A	*norpA*	**stimulated by light, contributes to the rapid temporal response of photoreceptor cells**	[7]
Rhodopsin 2	*Rh2*	**one of the four ocellar visual pigments in the major class of photoreceptor cells of the retina**	[5]

References

1. Orem NR, Xia L, Dolph PJ: **An essential role for endocytosis of rhodopsin through interaction of visual arrestin with the AP-2 adaptor.** *Journal of cell science* 2006, 119(15):3141-3148.
2. Yang Y, Ballinger D: **Mutations in calphotin, the gene encoding a** *Drosophila* **photoreceptor cell-specific calcium-binding protein, reveal roles in cellular morphogenesis and survival.** *Genetics* 1994, 138(2):413-421.
3. Zelhof AC, Hardy RW, Becker A, Zuker CS: **Transforming the architecture of compound eyes.** *Nature* 2006, 443(7112):696-699.
4. Dolph PJ, Man-Son-Hing H, Yarfitzt S, Colley NJ, Running Deer J, Spencer M, Hurley JB, Zuker CS: **An eye-specific G[beta] subunit essential for termination of the phototransduction cascade.** *Nature* 1994, 370(6484):59-61.
5. Zuker CS: **The biology of vision of *Drosophila*.** *Proceedings of the National Academy of Sciences* 1996, 93(2):571-576.
6. Porter JA, Montell C: **Distinct roles of the *Drosophila* ninaC kinase and myosin domains revealed by systematic mutagenesis.** *The Journal of cell biology* 1993, 122(3):601-612.
7. Deer JLR, Hurley JB, Yarfitz SL: **G protein control of** *Drosophila* **photoreceptor phospholipase C.** *Journal of Biological Chemistry* 1995, 270(21):12623-12628.
