# Supplementary material for: RNA-sequencing elucidates the regulation of behavioural transitions associated with the mating process in honey bee queens
Source: BMC Genomics. 2015 Jul 31;16:563. doi: 10.1186/s12864-015-1750-7 (PMC4521450; doi:10.1186/s12864-015-1750-7)
Supplement: Additional file 7: — Results of comparative studies. [file 12864_2015_1750_MOESM7_ESM.pdf]

Additional file 7. Results of comparative studies

We found recurrent themes in the comparative analyses with previous studies analysing similar biological processes, but also different magnitude of overlap for genes that were differentially regulated in each study.

Kocher et al. [8]. In this study the authors examined brain gene expression in virgin, mated and egg-laying honey bee queens using microarrays. The overlap of whole datasets between the two studies produced a set of 58 common genes that were differentially expressed (Additional file 2): this result was not statistically significant (hypergeometric test: representation factor: 1.0, P-value = 0.475). However, a gene ontology analysis on this small set of genes revealed that they were associated with 3 significantly overrepresented GO terms: response to other organism (P-value = 0.007, 4 genes), oxidation reduction (P-value = 0.008, 8 genes) and antimicrobial humoral response (P-value = 0.019, 3 genes).

When we performed an overlap of the gene lists for the mated vs. virgin comparison only, there was a set of 24 genes in common between the two studies (Additional file 3): again, this result was not statistically significant (hypergeometric test: representation factor: 0.9, P-value = 0.262).

Kocher et al. [9]. Here the authors examined the effects of mating and instrumental insemination with saline or semen on gene expression in the brains of honey bee queens using microarrays. A set of 58 genes were significantly differentially expressed in both studies (Additional file 2): this is more than expected by chance (hypergeometric test: representation factor: 1.7, P-value = 7.026^{-05}). GO terms associated with the common genes and significantly overrepresented included: humoral immune response (P-value = 0.031, 3 genes), lipid particle (P-value = 0.036, 5 genes) and transporter activity (P-value = 0.040, 9 genes).

The overlap analysis between the gene lists from the two studies for the mated vs. virgin comparison identified 11 genes that were in common (Additional file 4): however, this result was not statistically significant (hypergeometric test: representation factor: 1.0, P-value = 0.482).

Overall comparison with Kocher et al studies [8, 9]. In order to further characterize the major genes involved in the mating process and reproductive activation in the brain of honey bee queens, we identified those differentially expressed genes that were shared among whole datasets from this study, Kocher et al. [8] and Kocher et al. [9]. We found 15 genes that were significantly differentially expressed across the 3 studies (Figure 4). Several of these genes are described in *D. melanogaster* and play an interesting role for relevant biological processes: *Defensin (Def)* for immune response [10], *Desaturase 1 (desatl)* for mating behaviour [11], *Elongation factor 1a100E (EflalphalOOE)* for GTPase activity, *Epidermal stripes and patches (Esp)* for negative regulation of female receptivity [12], *Heat shock protein 83 (Hsp83)* for oogenesis, sleep and response to heat [13-15], *Kaz1-ORFB, lethal (2) essential for life (l(2)efl)* for embryo development and response to heat, *Myosin light chain 2 (Mlc2)* for flight activity [16], *Stretchin-Mlck (Strn-Mlck)* involved in myosin light chain kinase activity [17], *Odorant receptor 13a (Or13a)* for sensory perception of smell, *Ejaculatory bulb protein III (Peblll)* for response to virus [18] and*yellow-h* for melanin biosynthetic process [19].

Nino et al. [20]. In this study brain transcriptomic profile was evaluated in virgin honey bee queens, CO_2-treated and physically manipulated (i.e. exposed to CO_2 and sham-inseminated). There were 76 genes in common across the whole datasets from the two studies but this result was not statistically significant (hypergeometric test: representation factor: 1.0, P-value = 0.467).

Shared genes belonged to 1 metabolic pathway and 8 GO terms that were significantly overrepresented (Additional file 2). Among these, GO terms of particular interest were response to stimulus (P-value = 002, 15 genes), regulation of response to stimulus (P-value = 0.012, 3 genes), response to other organism (P-value = 0.002, 5 genes), immune response (P-value = 0.004, 5 genes) and regulation of antimicrobial humoral response (P-value = 0.012, 3 genes).

We also performed an overlap analysis with Nino et al. [20] for genes that were specifically different between CO_2-treated and virgin queens (Additional file 5). The analysis revealed that only 2 genes were shared across the two studies and this result was not statistically significant (hypergeometric test: representation factor: 1.1, P-value = 0.440).

Brito et al. [21]. Finally, we considered the list of 20 candidate genes implicated in ovary activation that Brito et al. [21] tested in their QPCR study for response to double narcosis with carbon dioxide in queens and workers. Of these genes, 7 were significantly differentially expressed in at least one comparison across our three treatment groups (Figure 5). Patterns of expression in 8-day-old queens were consistent for 5 genes across the two studies: *take-out-like (JHBP1), phosphoinositide- dependent kinase (PDK1)* and *ovary activation candidate 001 (OAC001)* were up- regulated while *myosin regulatory light chain 2 (Mlc-2)* and *SPARC* were down-regulated in CO_2-treated queens compared to virgin queens. *Midway (Mid)* and *vitellogenin (Vit)* had opposite trends instead: they were up-regulated in CO_2-treated queens in our study and down-regulated in Brito et al. [21].

References

1. Orem NR, Xia L, Dolph PJ: **An essential role for endocytosis of rhodopsin through interaction of visual arrestin with the AP-2 adaptor.** *Journal of cell science* 2006, 119(15):3141-3148.
2. Yang Y, Ballinger D: **Mutations in calphotin, the gene encoding a *Drosophila* photoreceptor cell-specific calcium-binding protein, reveal roles in cellular morphogenesis and survival.** *Genetics* 1994, 138(2):413-421.
3. Zelhof AC, Hardy RW, Becker A, Zuker CS: **Transforming the architecture of compound eyes.** *Nature* 2006, 443(7112):696-699.
4. Dolph PJ, Man-Son-Hing H, Yarfitzt S, Colley NJ, Running Deer J, Spencer M, Hurley JB, Zuker CS: **An eye-specific G[beta] subunit essential for termination of the phototransduction cascade.** *Nature* 1994, 370(6484):59-61.
5. Zuker CS: **The biology of vision of *Drosophila.*** *Proceedings of the National Academy of Sciences* 1996, 93(2):571-576.
6. Porter JA, Montell C: **Distinct roles of the *Drosophila* ninaC kinase and myosin domains revealed by systematic mutagenesis.** *The Journal of cell biology* 1993, 122(3):601-612.
7. Deer JLR, Hurley JB, Yarfitz SL: **G protein control of *Drosophila* photoreceptor phospholipase C.** *Journal of Biological Chemistry* 1995, 270(21):12623-12628.
8. Kocher SD, Richard F-J, Tarpy DR, Grozinger CM: **Genomic analysis of post-mating changes in the honey bee queen *(Apis mellifera).*** *BMC genomics* 2008, 9(1):232.
9. Kocher S, Tarpy D, Grozinger C: **The effects of mating and instrumental insemination on queen honey bee flight behaviour and gene expression.** *Insect molecular biology* 2010, 19(2):153-162.
10. Imler J, Bulet P: **Antimicrobial peptides in *Drosophila:* structures, activities and gene regulation.** 2005.
11. Bousquet F, Nojima T, Houot B, Chauvel I, Chaudy S, Dupas S, Yamamoto D, Ferveur J-F: **Expression of a desaturase gene, desatl, in neural and nonneural tissues separately affects perception and emission of sex pheromones in *Drosophila.*** *Proceedings of the National Academy of Sciences* 2012, 109(1):249-254.
12. Findlay GD, Sitnik JL, Wang W, Aquadro CF, Clark NL, Wolfner MF: **Evolutionary rate covariation identifies new members of a protein network required for *Drosophila melanogaster* female post-mating responses.** *PLoS genetics* 2014, 10(1):e1004108.
13. Pisa V, Cozzolino M, Gargiulo S, Ottone C, Piccioni F, Monti M, Gigliotti S, Talamo F, Graziani F, Pucci P: **The molecular chaperone Hsp90 is a component of the cap-binding complex and interacts with the translational repressor Cup during *Drosophila*** oogenesis. *Gene* 2009, 432(1):67-74.
14. Shaw PJ, Tononi G, Greenspan RJ, Robinson DF: **Stress response genes protect against lethal effects of sleep deprivation in *Drosophila.*** *Nature* 2002, 417(6886):287-291.

17. Neal SJ, Karunanithi S, Best A, So AK, Tanguay RM, Atwood HL, Westwood JT: **Thermoprotection of synaptic transmission in a** *Drosophila* heat shock factor mutant is accompanied by increased expression of Hsp83 and DnaJ-1. *Physiological Genomics* 2006, 25(3):493-501.
16. Miller MS, Farman GP, Braddock JM, Soto-Adames FN, Irving TC, Vigoreaux JO, Maughan DW: **Regulatory light chain phosphorylation and N-terminal extension increase cross-bridge binding and power output in *Drosophila* at in vivo myofilament lattice spacing.** *Biophysical Journal* 2011, 100(7):1737-1746.
17. Champagne MB, Edwards KA, Erickson HP, Kiehart DP: ***Drosophila* Stretchin-MLCK is a novel member of the Titin/Myosin light chain kinase family.** *Journal of Molecular Biology* 2000, 300(4):759-777.
18. Sabatier L, Jouanguy E, Dostert C, Zachary D, Dimarcq JL, Bulet P, Imler JL: **Pherokine-and-3.** *European Journal of Biochemistry* 2003, 270(16):3398-3407.
19. Gaudet P, Livstone M, Thomas P, The Reference Genome P: **Annotation inferences using phylogenetic trees.** In.; 2010.
20. Nino E, Tarpy D, Grozinger C: **Genome-wide analysis of brain transcriptional changes in honey bee *(Apis mellifera* L.) queens exposed to carbon dioxide and physical manipulation.** *Insect molecular biology* 2011, 20(3):387-398.
21. Brito R, McHale M, Oldroyd B: **Expression of genes related to reproduction and pollen foraging in honey bees *(Apis mellifera)* narcotized with carbon dioxide.** *Insect molecular biology* 2010, 19(4):451-461.
